# Development of Carrageenan and Starch-Based Bioplastics for Packaging Applications (Shopping Bags): Mechanical Characterization, Morphology, and Biodegradation

**DOI:** 10.1155/sci5/8879516

**Published:** 2025-10-22

**Authors:** Reni Giarni, Roni Sujarwadi, Yayat Iman Supriyatna, Indah Kurniasari, Maya Soraya, Renny Primasari Gustia Putri, Hendrawan Laksono, Heri Purwoto

**Affiliations:** ^1^Research Center for Polymer Technology, National Research and Innovation Agency (BRIN), KST BJ Habibie, Setu, South Tangerang 15314, Banten, Indonesia; ^2^Material and Structural Mechanics Laboratory, National Research and Innovation Agency (BRIN), KST BJ Habibie, Setu, South Tangerang 15314, Banten, Indonesia; ^3^Research Center for Mineral Technology, National Research and Innovation Agency (BRIN), KST Iskandar Zulkarnain, Tanjung Bintang, South Lampung 35361, Indonesia; ^4^Research Center for Process Technology, National Research and Innovation Agency (BRIN), KST BJ Habibie, Setu, South Tangerang 15314, Banten, Indonesia

**Keywords:** ASTM G21, bioplastic, carrageenan, starch, tensile strength

## Abstract

One approach to mitigating plastic pollution is the development of biodegradable plastic materials, such as bioplastics. Bioplastics are packaging materials that can be naturally degraded by microorganisms. In this study, bioplastics were produced using natural polymer compounds, specifically carrageenan and starch. The combination of starch and carrageenan was investigated to develop bioplastic packaging (shopping bags) with improved properties. This study aimed to evaluate the effect of incorporating different types of starch (corn, sago, and cassava) into carrageenan-based bioplastics on their physicochemical and mechanical characteristics. The research involved the fabrication of bioplastics using a combination of carrageenan and various starches (corn, sago, and cassava), followed by characterization, including moisture content, thickness, tensile strength, elongation at break, functional group analysis using Fourier transform infrared (FTIR) spectroscopy, and surface morphology analysis using scanning electron microscopy–energy-dispersive X-ray spectroscopy (SEM–EDS). Additionally, water vapor transmission rate (WVTR), thermogravimetric analysis (TGA), and biodegradation tests were conducted following the ASTM G21 standard. The results indicated that starch variation did not significantly affect the mechanical properties, morphology, or biodegradation characteristics of the carrageenan–starch bioplastics.

## 1. Introduction

Plastic is a synthetic polymer from petroleum raw materials. Plastic is widely used as packaging to meet daily needs. However, plastic causes problems for the environment because plastic waste cannot be broken down naturally by microbes. The total waste in Indonesia reported by the Ministry of Environment and Forestry in 2019 is estimated at 68 million tons, of which 9.52 million tons is plastic waste or 14% of the total waste produced. Apart from that, Indonesia is the second country in the world after China in producing plastic waste in waters ranging between 0.48 and 1.29 million metric tons per year. Some efforts to overcome pollution due to plastic waste have been carried out, such as the 3R program (reduce, reuse, and recycle), but these efforts have not optimally addressed environmental pollution that comes from plastic waste. Another effort to overcome this environmental problem is by developing plastic materials that are easily decomposed, namely, bioplastics. Bioplastics are packaging that can be degraded naturally by microorganisms. The materials used in making bioplastics are natural polymer compounds such as carrageenan and starch. Moreover, microplastic pollution is currently a global problem affecting the world. One contributor to microplastic pollution is the use of single-use plastics, such as shopping bags. Therefore, this research aims to develop bioplastics for use in shopping bags.

Currently, shopping bags made from cassava starch are widely sold commercially. Therefore, this study aims to try making shopping bags from carrageenan. One reason for making bioplastics from carrageenan is because seaweed is a widely cultivated product. Indonesia is one of the countries that supplies seaweed to the international market. Carrageenan is an extract that can be obtained from red seaweed (Rhodophyceae), which is a polysaccharide that is thick and can form a gel. Additionally, it is a sulfated polygalactan containing 15%–40% sulfate esters. Carrageenan is also formed by the alternative units d-galactose and 3,6-anhydro-D-galactose, combined with 1,3 and 1,4-glycosidic bonds. There are several types of carrageenan, such as kappa, iota, and lambda. Kappa carrageenan shows excellent properties for forming gels and membranes, which have better mechanical properties than other types of carrageenan [1].

Carrageenan is a linear polysaccharide and a galactan molecule with galactose as the main units. Pure carrageenan cannot be made into plastic and requires plasticizers. Plasticizers are polyfunctional combinations; for example, polyol (sorbitol) can be used as a plasticizer because it can form cross-links with carrageenan molecules, resulting in flexible properties and reducing the level of brittleness of the resulting film [2]. Making bioplastics from seaweed or carrageenan has been carried out, as done by Gebrina et al. [3] by making packaging using a carrageenan base. However, making bioplastic from carrageenan is considered too expensive for a shopping bag. So it is necessary to add other natural ingredients or polysaccharides, which are cheaper and more abundant, namely, starch.

Starch is the most abundant and renewable polysaccharide in plants, biodegradable, produced in large quantities at low cost, easy to handle, and exhibits thermoplastic behavior. Starch can be extracted from grains (e.g., corn, wheat, or rice), from tubers (e.g., potatoes, cassava, or cassava), or even from nuts (e.g., cashew nuts). Starch granules are insoluble in cold water and consist of two types of glucose polymers: amylose (a linear polymer consisting of about 20% w/w of starch granules) and amylopectin (a branched polymer). Starch properties depend directly on the botanical source, grain size distribution and morphology, genotype, amylose/amylopectin ratio, and other factors such as composition, pH, and chemical modification [4].

Most starch is composed of two major polysaccharides: amylose and amylopectin. Amylose is a primarily linear polysaccharide of a-1,4-linked D-glucopyranose with a few branches of a-1,6 linkages. Amylopectin, however, is a highly branched polysaccharide with a-1,4-linked linear chains of different lengths connected by approximately 5% a-1,6 branch linkages. The two main components of starch have distinctly different properties. In an aqueous dispersion, amylose has a greater tendency to recrystallize (known as retrogradation), forms strong gels and films, and develops a dark-blue color after complexing with iodine. In contrast, amylopectin retrogrades more slowly, forms weak gels and brittle films, and displays a purple to red color after complexing with iodine. The amylose content and branch chain length distribution of amylopectin directly determine many functional properties of starch, including gelatinization, pasting, gelling, and retrogradation/syneresis [5].

Starch, the major energy reserve in green plants, is commonly found in seeds (e.g., cereal grains and pulses), tubers (e.g., potato), roots (e.g., cassava and sweet potato), fruits (e.g., banana and squash), stems (e.g., sago), and leaves (e.g., tobacco). Starch is the predominant component of cereal grains, pulses, and tuber and root crops. For example, milled rice kernels contain up to 90% starch (dry basis, db), maize kernels up to 80% starch (dry basis, db), and pulse grains up to 53% starch (dry basis, db). Starch molecules are effectively organized in semicrystalline granules, which has a density of around 1.5 g/cm^3^. The greater density of starch granules than that of water allows for easy isolation and purification of starch by gravity sedimentation. The semicrystalline structure of starch granules maintains the granular integrity and prevents the dispersion of the granules in water at ambient temperature. Heating starch in the presence of water (as a plasticizer) can gelatinize and disperse native starch granules. Starches from different botanical origins display characteristic gelatinization properties, reflecting distinct structures of starch molecules, and the organization of double-helical crystalline structures inside starch granules. Without the presence of water or other plasticizers, starch does not gelatinize at high temperature. Instead, it will decompose when the temperature reaches 250°C and beyond [5].

Corn is the third most important grain crop in the world after wheat and rice. It serves as a primary energy source in livestock feed. Corn is processed into a multitude of food and industrial products, including starch, sweeteners, corn oil, beverages and industrial alcohol, and biofuel. Corn starch is used in a broad variety of foodstuffs and applications. Basic corn starches have a small amount of protein (0.35%), lipid (0.8%), ash, and > 98% of two polysaccharides, namely, amylose and amylopectin. Starch comes from plant sources that are insoluble in water, and at room temperature, it is in the form of granules. Usually, corn and waxy maize starch granules differ in their size from 2 to 30 mm; most fall in the range of 12–15 mm. They also differ in shape, appearing as cross sections of polygons [6].

Sago starch is produced from the trunk of true sago palm (Metroxylon sagu), which is native to Southeast Asia. Major growers of sago palm include Malaysia, Indonesia, and Papua New Guinea. The total starch content of sago pith was very high (e.g., 82%). Traditionally, starch is commonly extracted from the pith using water to make slurry with pith pieces. The slurry is passed through a series of sieves to remove the impurities such as fibers. The resulting starch cake is dried to obtain pure sago starch. The chemical composition of starch is critical for functional properties. The amylose contents of sago starch ranged from 21.4% to 30.0%. It should be stressed that the amylose content of starch can also be affected by the quantification methods used. Sago starches extracted from the palm of four different developing stages had a similar amylose content. Previous studies showed that sago starch tended to have very low levels of minor components such as protein (∼0.3%), lipids (∼0.1%), fiber (∼0.3%), and ash (∼0.4%). There was a low level (e.g., 86–97 ppm) of phosphorus-containing compounds mostly binding to C6 of the glucosyl residues in sago starch [7].

Cassava starch or cassava is widely used as feed, food, energy sources, and as auxiliary materials in various industries for various purposes. In general, the process of making cassava includes peeling the cassava skin, washing, grating, squeezing or extraction, starch sedimentation, drying, and packaging. The physicochemical characteristics of cassava are determined by the main components of cassava. Amylose and amylopectin are important components that form the basic structure of starch and can affect the physicochemical characteristics of the resulting starch. The difference in the ratio between amylose and amylopectin in starch affects its physicochemical properties [8].

Bioplastics consisting of starch and carrageenan are hydrophilic and have less elastic properties, so additional materials need to be added to improve their mechanical characteristics, such as plasticizers. Glycerol plasticizers have the ability to reduce internal hydrogen bonds and increase the distance between polymer molecules [9]. Plasticizers are added to increase film flexibility. The greater the use of plasticizers, the greater the percentage of elongation of a film. Commonly used plasticizers are glycerol, sorbitol, and polyethylene glycol. Glycerol is hydrophilic so it is easily dissolved during the formation of a film solution. In addition, glycerol has the advantage of being colorless, so it does not cause discoloration of bioplastics.

**Figure 1:**
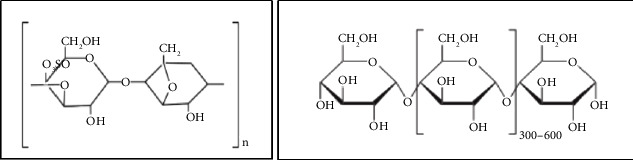
Structure of carrageenan and starch.

The purpose of this study was to determine the effect of several types of starch (corn, sago, cassava) on the mechanical, morphological, and biodegradability characteristics of carrageenan bioplastics. From this study, it is expected to obtain a bioplastic formula based on carrageenan and starch, which has two different polysaccharide structures, which can be seen in Figure 1 in order to apply it as a packaging (shopping bag) that meets the standards.

## 2. Materials and Methods

### 2.1. Materials

The materials utilized in this study comprised kappa carrageenan from PT. Indoflora Cipta Mandiri, Malang, cassava flour “Rose Brand” produced by PT. Budi Starch & Sweetener Tbk, Lampung, Indonesia, sago flour “Sapapua” produced by PD. Harsindo United Internusa, Tangerang, Indonesia, corn flour “maizenaku” produced by Egafood, Jakarta, Indonesia, glycerol, distilled water, rubber, technical alcohol, test molds from InaCC F46 (*Aspergillus niger* Van Tremor), InaCC F158 (*Penicillium* sp), InaCC F157 (*Chaetomium globosum* Kunze ex Fr.), InaCC F115 (*Trichoderma harzianum* Rifai), NSA/nutrient salt agar (KH_2_PO_4_, MgSO_4_·7H_2_O, NH_4_NO_3_, NaCl, FeSO_4_·7H_2_O, ZnSO_4_·7H_2_O, MnSO_4_·H_2_O, agar, and K_2_HPO_4_), filter paper (positive control), and polyethylene (negative control).

### 2.2. Tools

Experimental procedures involved the use of essential equipment, including hotplate stirrer, beaker glass, oven, glass mold, spatula, siever, magnetic stirrer, analytical balance, Universal Testing Machine AT-U2001, FTIR (Fourier transform infrared spectroscopy) Bruker-Tensor II, FE-SEM (field emission-scanning electron microscopy) Thermo Scientific Quattro S, simultaneous thermal analyzer (STA 6000), silica gel, porcelain cup, desiccator, autoclave, laminar air flow, Bunsen burner, incubator, micropipette, 200-μL tips, Erlenmeyer flask, test tube, stopper, disposable petri dish, and tweezers.

### 2.3. Bioplastics Production

The making of bioplastics is done by mixing 1% carrageenan and 1% flour (corn, sago, and cassava) and 2% glycerol. First, the flour (corn, sago, cassava) is dissolved in distilled water until dissolved (clear and thick), by heating and stirring. After the starch is dissolved, carrageenan is added until dissolved (temperature 70°C–85°C). Finally, glycerol is added and stirred for 5 min. The mixture is strained, poured into a glass mold, and dried in an oven at a temperature of around 35°C for 24 h. Bioplastics are ready to be characterized.

### 2.4. Mechanical Test

Mechanical properties were evaluated through a series of standardized tests. Tensile strength and elongation at break were measured using the ASTM D882 method to determine the material's resistance to mechanical stress and its elasticity. Thickness measurements were taken using a precision caliper, ensuring accuracy in micrometer-scale readings. The moisture content was analyzed with a digital moisture analyzer to assess the water retention properties of the bioplastics, which can significantly impact mechanical performance.

### 2.5. Functional Group Analysis

Functional group characterization of the bioplastic sheets was performed using FTIR spectroscopy. Spectral data were collected in the range of 4000–400 cm^−1^, enabling the identification of the active functional groups present in the samples. This analysis provided insights into the chemical composition and interactions of carrageenan and starch within the bioplastic matrix, crucial for understanding their structural and functional attributes.

### 2.6. Amylose Content Analysis

100 mg of flour was placed in a test tube and then 1 mL of 95% ethanol and 9 mL of 1 N NaOH were added. The mixture was heated in boiling water (95°C) for 10 min until a gel was formed, and then, the entire gel was transferred into a 100-mL measuring flask. Water was added to the gel and shaken and then adjusted to 100 mL with water. A total of 5 mL of sample solution was put into a 100-mL measuring flask and 1 mL of 1 N acetic acid, 2 mL of 0.01 N iodine solution (gradually), and distilled water were added to the mark and shaken. The mixture was heated with a water bath at 30°C for 20 min and then the absorbance was measured with UV–vis spectrophotometer at a wavelength of 620 nm. The absorbance obtained was plotted on a standard curve to obtain the amylose concentration of the sample.

### 2.7. Water Vapor Transmission Rate (WVTR) Test

WVTR is the rate at which water vapor seeps in through a plastic film at a constant temperature and relative humidity. The method used to measure the WVTR is the ASTM E96-01 (2001) method. The plastic film sheet is then glued onto a container containing 20 g of silica gel. The bioplastic is cut into a circle and then conditioned in a desiccator containing saturated NaCl with a humidity of 75% and a temperature of 25°C. The container is weighed every hour for 5 h, and then the data are calculated using the following equation:

(1) $$\begin{array}{c} \text{WVTR}=\frac{n}{\text{tA}}, \end{array}$$

where *n* = the difference in weight gain (g), *t* = the difference in time used (5 h), and *A* = the area of edible film (m^2^)/diameter of the petri dish.

### 2.8. Thermogravimetric Analysis (TGA)

TGA was performed using a simultaneous thermal analyzer (STA 6000) to analyze the thermal stability of bioplastics. About 13 mg sample of bioplastic was used in the TGA measurements. The sample will be heated from 25°C to 550°C with a temperature increase rate of 10°C/min until it decreases in mass in a nitrogen flow (20 mL/min).

### 2.9. Plastic Biodegradation Test (ASTM G21)

The biodegradation test was conducted using the ASTM G21 method, which is a biodegradation test of plastic materials using mold. Assessment references based on ASTM G21-13 are shown in Table 1.

## 3. Results and Discussion

The making process of bioplastics basically uses the principle of gelatinization by adding a certain amount of water and heating it at a high temperature. When a certain amount of water is added to starch and heated at a high temperature, the starch particles will absorb water and swell. Starch has a gelatinization temperature ranging from 70°C–90°C, while the gelatinization temperature of carrageenan is 80°C. This study used a gelatinization temperature for starch and carrageenan in the temperature range of 70°C–85°C. According to McGrane et al. [10], during gelatinization, amylose molecules form intramolecular and intermolecular hydrogen bonds, which are crucial for the gel's structural integrity. In the presence of water, intermolecular hydrogen bonds dominate, leading to the formation of strong elastic gels. Amylose and amylopectin physically form intermolecular and intramolecular cross-links to form a larger macromolecular network in making gels. According to Pujawati et al. [9], one way to improve the mechanical properties of bioplastics is to combine starch and carrageenan materials so that they will produce cross-links.

According to Suryanto et al. [11], the interaction between starch and carrageenan determines the behavior of the constituent polymer phases, both phase separation and miscibility. Starch and carrageenan have good miscibility because carrageenan has a double helix structure that traps starch in a circular structure and acts as a protector of starch molecules. The strong interaction between starch and carrageenan produces a structure with high crystallinity, which strengthens the integrity of the bioplastic polymer chain.

**Figure 2:**
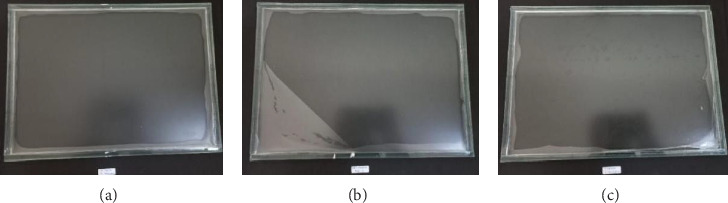
Bioplastics in molds ((a) corn starch–carrageenan, (b) sago starch–carrageenan, and (c) cassava–carrageenan).

**Figure 3:**
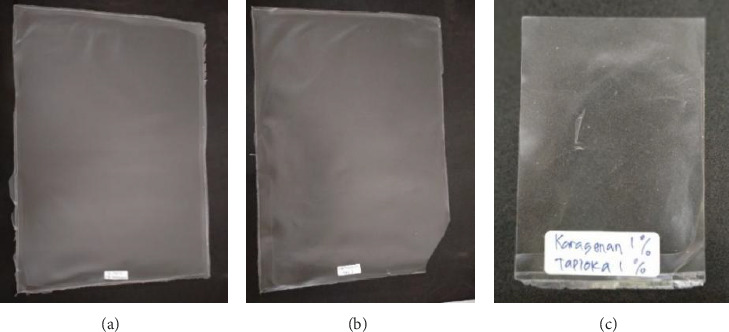
Bioplastics ((a) carrageenan–corn starch, (b) carrageenan–sago starch, and (c) carrageenan–cassava).

Figure 2 shows bioplastics made from carrageenan and several types of starch, namely, corn starch, sago, and cassava, which were then characterized. Based on their physical appearance, the three bioplastics produced appear clear to slightly opaque. This appearance is clearly visible in Figure 3, which shows the bioplastic product being released from its mold.

To determine whether or not there is an effect of starch variations (sago, corn, and cassava) used on the characteristics of bioplastics (tensile strength, elongation at break, thickness, water content, and WVTR values), a statistical test was carried out using the ANOVA test. The variation in starch is basically due to the difference in the amount of amylose contained in sago, corn, and carrageenan starch. The following are the results of the analysis of the amylose content of each starch used: 28.54% for sago starch, 27.61% for corn starch, and 26.59% for cassava starch.

### 3.1. Equilibrium Degree of Swelling

Tensile strength is an important parameter that affects the mechanical properties of bioplastics. Tensile strength is the maximum tensile stress of the sample before breaking; bioplastics with the highest tensile strength value can withstand physical damage during food packaging. Based on the results of the ANOVA test, a significant value of > 0.05 (*p* value = 0.148) was obtained, which means that there is no effect of the starch variation on the tensile strength value of carrageenan–starch bioplastics.  Table 2 shows that the tensile strength values of carrageenan–starch bioplastics are 9.243 MPa for carrageenan–sago, 10.104 MPa for carrageenan–corn, and 7.745 MPa for carrageenan–cassava. This can be caused because the amylose content of the three has almost the same value. The results of the analysis of the amylose content of each starch used are as follows. 28.54% for sago starch, 27.61% for corn starch, and 26.59% for cassava starch. According to Hirpara and Dabhi [12], it is stated that a high amylose content shows a higher tensile strength value. This is because amylose and amylopectin molecules contribute to the mechanical properties of starch. Amylopectin is a branched glucose polymer, while amylose is a linear glucose polymer. Cross-linking agents, such as glutaraldehyde, can further influence the crystallinity of amylose-rich films. While cross-linking generally reduces crystallinity by hindering the regular packing of polymer chains, it simultaneously enhances tensile strength by forming a more robust network [13]. High-amylose starches tend to form more crystalline structures compared to amylopectin-rich starches. This is because amylose can align more easily into ordered structures, which enhances the crystallinity of the films. For instance, dual-modified high-amylose maize starches showed increased crystallinity, which is crucial for improving mechanical properties [14]. Conversely, if bioplastics have high amylopectin, their tensile strength is weak. This is explained by Tan et al. [15] that the branched amylopectin structure creates distances between polymer chains, which results in weaker hydrogen bonds between the polymer chains. However, when compared to commercial bioplastics (shopping bags), which have a tensile strength value of 10,052 MPa, the carrageenan–starch bioplastic in the study had almost the same tensile strength value.

### 3.2. Elongation at Break

Elongation at break is the percentage change in the length of the bioplastic when pulled to break. Based on the results of the ANOVA test, a significant value of > 0.05 was obtained (*p* value = 0.1), which means that there is no effect of the starch variation on the elongation at break value of bioplastic.  Table 2 shows that the elongation at break value of the bioplastic is 26.74% for carrageenan–sago starch, 36.44% for carrageenan–corn starch, and 25.13% for carrageenan–cassava. This can be caused because the amylose content of the three starch has almost the same value. It is known that the amylose content in the starch used is as follows: 28.54% for sago starch, 27.61% for corn starch, and 26.59% for cassava starch. According to Hirpara and Dabhi [12], increasing the amylose content correlates with decreasing elongation at break. This is because high amylose causes strong interactions between starch molecules. The bonds that occur between starch molecules are tighter and more compact, causing the film to become strong and increasingly difficult to elongate. Research conducted by Szlachetka et al. [16] showed that film thickness can significantly impact tensile strength and elasticity. However, when compared to commercial bioplastics (shopping bags), which have an elongation at break value of 275.68%, the carrageenan–starch bioplastic in the study had a much smaller elongation at break value.

### 3.3. Thickness

The thickness of a bioplastic can be influenced by the amount of dissolved solids and the surface area of the bioplastic mold used. Based on the results of the ANOVA test, a significant value of > 0.05 (*p* value = 0.943) was obtained, which means that there is no effect of the starch variation on the thickness value of the carrageenan–starch bioplastic.  Table 2 shows that carrageenan–starch bioplastics have the same thickness, namely, 0.126 mm for carrageenan–sago starch, 0.127 mm for carrageenan–corn starch, and 0.128 mm for carrageenan–cassava. Horn et al. [17] stated that the use of starch with a high amylopectin content significantly reduces the film thickness, or conversely, a low amylose content will increase the thickness; it was also added that the film thickness was determined to calculate the water vapor permeability and tensile properties of the film. A higher film thickness indicates higher resistance, gas permeability, and mechanical properties. According to Borges et al. [18], thickness is important to evaluate the film homogeneity and is also related to strength and barrier properties (hydrophobicity). However, when compared to commercial bioplastics (shopping bags), which have a thickness value of 0.049 mm, the carrageenan–starch bioplastic in the study had a much greater thickness.

### 3.4. Moisture Content

The water content of a film depends on its composition and can also indicate biodegradability. Based on the results of the ANOVA test, a significant value of > 0.05 was obtained (*p* value = 0.437), which means that there is no effect of the starch variation on the water content of carrageenan–starch bioplastics.  Table 2 shows that the water content of bioplastics is 27.32% for carrageenan–sago starch, 26.43% for carrageenan–corn starch, and 29.81% for cassava–carrageenan. When compared to commercial bioplastics (shopping bags), which have a water content of 29.48%, the carrageenan–starch bioplastic in the study has almost the same water content.

### 3.5. WVTR

WVTR testing is carried out to determine how much water vapor can penetrate the bioplastic film layer. The factor that most affects the rate of water vapor transmission is the thickness of the film. The higher the thickness value, the stiffer and harder the film will be so that the ability to hold water vapor and gas will increase. WVTR is the constant rate of water vapor transmission through a number of unit areas under certain temperature and humidity conditions. From the resulting WVTR value, the amount of water vapor absorbed or released from a packaged product during storage can be estimated. A high water vapor transmission value indicates that the film is easier to pass through by water vapor. Glycerol has hydrophilic properties so that the rate of water vapor transmission will increase as the concentration of glycerol used increases. This is because glycerol has the ability to bind water so that it can increase the humidity of the film and also the rate of water vapor transmission. The rate of water vapor transmission of a material is also influenced by the concentration of plasticizer, environmental conditions, and the properties of chemicals that are hydrophilic and hydrophobic.

Based on the results of the ANOVA test, a significant value of > 0.05 was obtained (*p* value = 0.619), which means that there is no effect of the starch variation on the WVTR value of carrageenan–starch bioplastics.  Table 2 shows that the WVTR values of carrageenan–starch bioplastics have almost the same values. The WVTR value of bioplastics is 0.1815 g/hour·m^2^ for carrageenan–sago starch, 0.2041 g/hour·m^2^ for carrageenan–corn, and 0.1999 g/hour·m^2^ for carrageenan–cassava. Horn et al. [17] stated that films with a high amylopectin content produce films with low water vapor permeability (WVTR). However, when compared to commercial bioplastics (shopping bags), which have a WVTR value of 0.1344 g/hour·m^2^, the carrageenan–starch bioplastic in the study has almost the same WVTR value.

### 3.6. FTIR Analysis

FTIR analysis is performed to identify functional groups contained in bioplastics. The basic principle of IR spectrophotometric analysis is the absorption of electromagnetic radiation by certain functional groups so that from the absorption spectrum that is read, we can find out what functional groups are contained in a compound. When infrared light is passed through a sample, a number of frequencies are absorbed by the sample and other frequencies are forwarded or transmitted without absorption. The percentage of absorbance with frequency has a relationship to produce an infrared spectrum. FTIR test results are given in Table 3.

**Figure 4:**
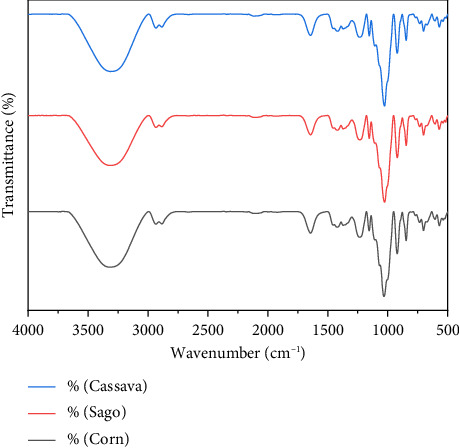
FTIR spectra of bioplastics.

Table 3 shows that both starch and carrageenan showed absorption at wave numbers 3580–3650 cm^−1^ (O–H bonds), 2800–3200 cm^−1^ and 1300–1475 cm^−1^ (C–H bonds), 1050–1300 cm^−1^ (C–O bonds), and 900–1200 cm^−1^ (C–O–C bonds). The distinguishing spectra are at wave numbers 700–900 cm^−1^, which are C–O–SO_3_ bonds, which are typical spectra of carrageenan. All bioplastics showed absorption at wave numbers above 3500 cm^−1^, which are –OH bonds. The addition of glycerol resulted in an increase in the wave number of the –OH functional group; this reaction occurs because glycerol has a chemical structure in the form of strong hydrogen bonds and forms intramolecular hydrogen bonds including with water molecules, resulting in hydrophilic plastic properties. According to Arzani et al. [19], the absorption spectrum on the FTIR spectrophotometer shows strong absorption at 1210–1260 cm^−1^, which is the absorption of the sulfate ester bond (S=O) for all types of carrageenan. The absorption area of 1010–1080 cm^−1^ shows the absorption of the glycosidic bond, and the absorption area of 840–850 shows the absorption of the galactose-4-phosphate bond; this galactose-4-phosphate bond shows the type of kappa carrageenan. According to Suryanto et al. [11], the wave number ranging from 800 to 1500 cm^−1^ is known as the kappa carrageenan fingerprint area. The wave number 2700–3000 cm^−1^ shows the stretching of the C–H group, and the range 3000–3600 cm^−1^ shows the stretching of the hydroxyl group (OH). Meanwhile, according to Flores et al. [20], the physical bond between starch and kappa carrageenan is likely caused by hydrogen bonds. The spectral results are shown in Figure 4.

**Figure 5:**
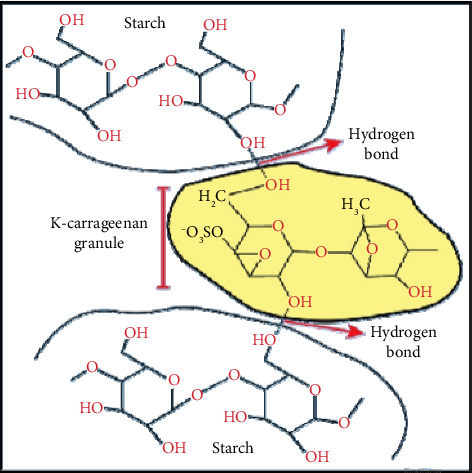
Hydrogen bonds between the starch matrix and kappa carrageenan [1].

According to Dawam Abdullah et al. [1], as an illustration, how hydrogen bonds occur between carrageenan and starch is shown in Figure 5.

### 3.7. SEM Analysis

**Figure 6:**
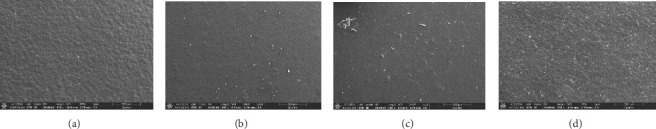
Bioplastic morphology with 150× magnification ((a) carrageenan–corn starch, (b) carrageenan–sago starch, (c) carrageenan–cassava, and (d) commercial bioplastic [shopping bags]).

**Figure 7:**
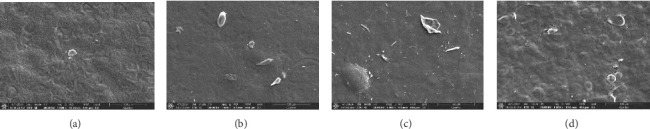
Bioplastic morphology with 1000× magnification ((a) carrageenan–corn starch, (b) carrageenan–sago starch, (c) carrageenan–cassava, and (d) commercial bioplastic [shopping bags]).

FE-SEM analysis of bioplastics aims to see the surface morphology of carrageenan–starch bioplastics and is equipped with a EDS (energy-dispersive spectrometer), which allows for the identification of elements contained in the material. Based on the results of SEM analysis (Figures 6 and 7), the surface of both carrageenan–starch bioplastics, corn–carrageenan, sago–carrageenan, and cassava–carrageenan, and commercial bioplastics (shopping bags) looks uneven or wavy. This is in accordance with the statement of Flores et al. [20] that when gelatinization occurs, heat and water weaken the inter- and intramolecular bonds in the film-forming solution. When the film-forming solution dries and cools, the molecular bonds become intertwined with each other, causing wrinkles and porous surfaces to the formation of bubble-like images. This observation can be attributed to the difference in density between carrageenan and starch. The physical bond between cassava starch and kappa carrageenan is likely caused by hydrogen bonds. According to Hirpara and Dabhi [12], the amylose network in the film can be observed by transmission electron microscopy, consisting of rigid strands and open pores and becoming opaque as the proportion of amylose decreases.

**Figure 8:**
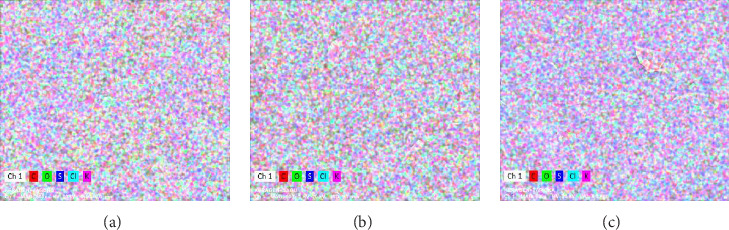
EDS analysis results of bioplastics ((a) carrageenan–corn starch, (b) carrageenan–sago starch, and (c) carrageenan-cassava).

Table 4 shows the results of the analysis of the content of elements in bioplastics using FE-SEM equipped with a EDS that bioplastics contain carbon (C), oxygen (O), and sulfur (S). This is because carrageenan, starch, and glycerol are compounds composed of C, H, O, and S (for carrageenan), while potassium (K) and chloride (Cl) are substances contained in the carrageenan used.  Figure 8 shows the distribution of sulfur (S) produced from sulfate esters derived from carrageenan. According to Dawam Abdullah et al. [1], carrageenan contains the element S, which is not found in starch, thus strengthening its mechanical properties.

### 3.8. TGA

TGA is used to determine the thermal stability of bioplastics. The TGA curve illustrates the degradation of bioplastics caused by temperature. When heat is applied to bioplastic, the temperature increases over time. It was found that the weight of the bioplastic decreases with increasing temperature. TGA curves can also be used as qualitative analysis by comparing the thermal stability of a material. The TGA curves indicate that the three bioplastics have almost the same thermal stability.

**Figure 9:**
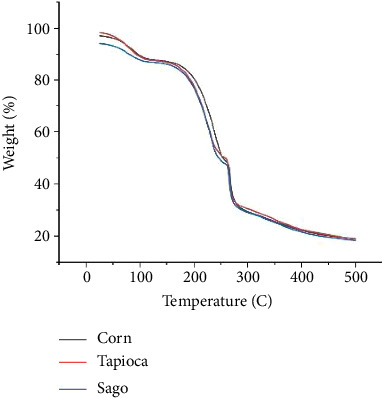
TGA curves of bioplastics.

Based on the TGA curve in Figure 9, the pattern of weight loss or decomposition of bioplastics related to temperature has almost the same curve pattern for corn–carrageenan, sago–carrageenan and tapioca–carrageenan bioplastics. Three distinct steps occurred during the thermal decomposition of bioplastics on the TGA curve. The first decomposition occurs in the temperature range of 50°C–150°C, caused by the release of water molecules from the bioplastic material. This is consistent with research by Mahardika et al. [21], who found that the first phase of film decomposition occurs at temperatures below 100°C, specifically in the range of 40°C–47°C, caused by the evaporation of water content from the film. The second decomposition occurs at temperatures of 150°C–250°C due to the evaporation of the plasticizer, glycerol. This is consistent with research by Sari et al. [22] and Mahardika et al. [21], who found that the evaporation of plasticizers, such as glycerol, sorbitol, and propylene glycol, occurs within a temperature range of 125°C–290°C. The third decomposition stage occurs at temperatures of 250°C–500°C, which is the decomposition process of starch and carrageenan. According to Abdullah et al. [23], degradation at temperatures of 250°C–400°C is caused by decarboxylation, depolymerization, and decomposition of glycosyl groups in the starch matrix. According to Sari et al. [22], starch decomposition occurs at temperatures ranging from 204.34°C to 433.21°C. At this stage, hydrogen groups are removed and the carbon chains of the starch depolymerize at 300°C. According to Amin et al. [24], starch bioplastic decomposition occurs at temperatures of 291°C and 303°C. According to Mahardika et al. [21], temperatures above 290°C produce the highest thermal decomposition rates, which can be attributed to the elimination of hydrogen functional groups, as well as degradation and depolymerization processes affecting the κ-carrageenan chain polymers. Decomposition of starch and carrageenan means that the chemical bonds in the starch and carrageenan molecules are broken, or the bonds in the structure of the starch and carrageenan molecules begin to break, causing changes in physical and chemical properties.

TGA is an analysis performed to determine the thermal resistance of a material. This thermal resistance is closely related to its mechanical properties. The higher the thermal resistance of a material, the higher its mechanical properties, or the higher its tensile strength, the higher its thermal resistance [22]. If the thermal resistance of carrageenan–starch bioplastic is associated with the tensile strength of the bioplastic, then the statement is correct. This is because based on the TGA curve, the three bioplastics have almost the same thermal resistance, and the tensile strength of the three bioplastics also statistically shows no significant difference between the three or has almost the same tensile strength.

### 3.9. Biodegradation Testing

Bioplastic biodegradation test is conducted using the ASTM G21 method. ASTM G21 is a biodegradation test of plastic materials against mold (fungi), which aims to determine the resistance of plastic materials to the growth of mold or fungi. Biodegradable plastic is a plastic that can be degraded by microorganisms such as bacteria, algae, fungi, and others. This method is designed to be applied to starch-based plastic samples. The principle of testing is to place the test object (bioplastic) on the surface of mineral salt agar in a petri dish that does not contain additional carbon. The test object and agar media are sprayed with a mixture of microorganisms that have been determined in each test. Organisms that are usually used as test organisms are those that have the ability to reproduce repeatedly and whose ability has been proven over a long period of time under laboratory conditions and in specific control and culture media. This test is evaluated based on the growth of microorganisms in the media used. The results of the biodegradability test can be seen qualitatively, by visually observing the growth of the test mold. If there is growth, it is indicated by the presence of mold mycelium attached to the surface of the bioplastic.

**Figure 10:**
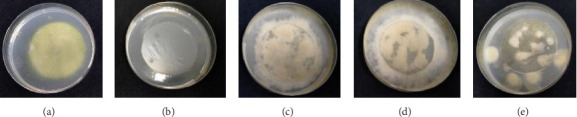
Biodegradation test results ((a) positive control [filter paper], (b) negative control [polyethylene], (c) carrageenan–corn starch, (d) carrageenan–sago starch, and (e) carrageenan–cassava).

Figure 10 shows that the positive control (filter paper) is completely filled with test mold mycelia on its surface, while the negative control (polyethylene plastic) has no mold growth at all on the surface. This shows that filter paper as a positive control can be a growth medium for test mold, in which case the growth of the positive control is 100% (maximum growth rate or level 4), while polyethylene as a negative control cannot be a growth medium for test mold. Based on the observations, the growth of test mold on carrageenan–starch bioplastic has a growth rate of more than 60% (Level 4), for carrageenan–corn starch, carrageenan–sago starch, and carrageenan–cassava bioplastics. This shows that the bioplastic tested can be used as a growth medium for test mold and can be biodegraded by microbes (biodegradable), so it is recommended as an environmentally friendly material.

After 28 days of incubation, mold growth was seen on the surface of the bioplastic. From the mold growth ranking table, it can be seen that mold growth on bioplastic is included in the highest ranking category, namely, the percentage of mold growth is more than 60% of the entire bioplastic surface. Thus, it can be concluded that the mold can use bioplastic as a food source for its growth. The test mold used in the ASTM G21 plastic biodegradation test is a mold that is generally found in the soil. The test mold used consists of *A. niger* Van Treghem (InaCC F46), *Penicillium* sp (InaCC F158), *C. globosum* Kunze ex Fr. (InaCC F157), and *T. harzianum* Rifai (InaCC F115). Mold that colonizes the surface of bioplastic and utilizes its polymer will show growth characteristics that can be observed directly through the growth of mycelia and hyphae. Mold, a type of fungus, is a heterotrophic organism that relies on organic compounds for nutrition. It absorbs nutrients from its environment through structures called hyphae and mycelium, storing energy in the form of glycogen. Molds are eukaryotic organisms that play a significant role in various ecological and industrial processes. They are essential decomposers in nature, breaking down organic matter and recycling nutrients, and are also utilized in food production and biotechnology [25–27].

The ability of fungi from the genera Aspergillus, Trichoderma, Penicillium, and Fusarium to degrade bioplastics is a promising area of research in the field of bioremediation. These fungi have shown potential in breaking down various types of synthetic plastics, which are otherwise resistant to degradation and pose significant environmental challenges [28–31]. Molds have enzymes that play an important role in breaking down bioplastic polymers. Enzymes that can hydrolyze starch consist of three groups. First, there is the enzyme α-amylase (α-1,4-glucan glucanohydrolase) or commonly called endoamylase. This enzyme hydrolyzes the α-1,4 glycosidic bonds in amylose and amylopectin randomly to produce dextrin and maltose. Furthermore, the product will be hydrolyzed by the glucogenic enzyme into glucose. Second, there is the enzyme α-amylase (α-1,4-glucan maltohydrolase) also called exoamylase. This enzyme hydrolyzes the main chain in starch. The third enzyme is glucoamylase, which plays a role in the process of depolymerizing glucose components into simpler chains. Some of the components that are often mentioned are CO_2_ and H_2_O.

Molds utilize complex organic polymers and convert them into simpler molecules by secreting degrading enzymes that will break the polymer chain and produce short chains, such as oligomers, dimers, and monomers, but the large molecular weight of bioplastics, three-dimensional structure, hydrophobic properties, and minimal functional groups greatly affect the microbial attack efforts on bioplastic polymers. Filamentous fungi are generally able to attach to hydrophobic surfaces by forming hydrophobic proteins. Extracellular enzymes are too large to penetrate deeper into the polymer material so they only work on the polymer surface. As a result, biodegradation of bioplastics is generally only a surface erosion process [32].

## 4. Conclusion

This study confirmed that carrageenan–starch-based bioplastics, incorporating different starch sources—corn, sago, and cassava—exhibited consistent mechanical properties, morphological characteristics, and biodegradability. The formulations demonstrated comparable tensile strength, elongation at break, thickness, and WVTRs, indicating their structural integrity and functional reliability across different starch types. Additionally, all bioplastics showed effective biodegradation, as evidenced by extensive fungal colonization, reinforcing their potential as environmentally sustainable packaging materials. The uniformity of these properties across starch variations suggests flexibility in raw material selection, ensuring the adaptability of carrageenan–starch bioplastics without compromising performance.

## Figures and Tables

**Table 1:** Plastic biodegradation level.

No.	Growth observation on specimens	Level
1	No growth	0
2	Minimal growth (< 10%)	1
3	Slight growth (10%–30%)	2
4	Moderate growth (30%–60%)	3
5	Maximum growth (60%–total)	4

**Table 2:** Bioplastic characteristics.

No	Bioplastic	Tensile strength (MPa)	Elongation at break (%)	Thickness (mm)	Moisture content (%)	WVTR (g/hr·m^2^)
1	Carrageenan–sago starch	9.243 ± 0.00	26.74 ± 0.00	0.126 ± 0.02	27.32 ± 3.44	0.1815 ± 0.03
2	Carrageenan–corn starch	10.104 ± 0.47	36.44 ± 6.16	0.127 ± 0.00	26.43 ± 1.02	0.2041 ± 0.02
3	Carrageenan–cassava	7.745 ± 1.41	25.13 ± 1.82	0.128 ± 0.01	29.81 ± 1.95	0.1999 ± 0.02
4	Commercial bioplastic (shopping bags)	10.052 ± 1.04	275.69 ± 54.20	0.049 ± 0.00	29.48 ± 0.00	0.1344 ± 0.00

**Table 3:** Fourier transform infrared spectroscopy spectra.

Wave number (cm^−1^)			Functional group	Reference wave number (cm^−1^)
Carrageenan–corn starch	Carrageenan–sago starch	Carrageenan–cassava		
3311.53	3309.76	3305.19	OH	3000–3600
2934.59	2933.77	2933.95	C–H	2700–3000
2884.59	2884.80	2884.51	C–H	2700–3000
1233.03	1232.70	1232.78	Sulfate ester bond (S=O)	1210–1260
1030.65	1027.18	1027.77	Glycosidic bond (C–O–C)	1010–1080
921.06	920.32	920.90	3,6-Anhydro-D-galactose (C–O)	928–933
846.19	845.59	846.07	D-Galactose-4-sulfate (C–O–SO_3_)	840–850

**Table 4:** EDS analysis results of bioplastic.

Element	Carrageenan–corn starch (%)	Carrageenan–sago starch (%)	Carrageenan–cassava (%)
C	51.47	66.88	58.80
O	31.47	27.67	29.88
S	2.57	1.18	2.36
K	4.75	2.58	4.86
Cl	1.52	1.12	1.62

## Data Availability

Data are available on request from the authors.
